# Coronary Artery Perforation During PCI: Mechanisms, Management, and Outcomes From a 10-Year Experience

**DOI:** 10.1016/j.jaccas.2025.105068

**Published:** 2025-09-24

**Authors:** Ravi Sankar Tulluru, Karthik Gopinath, Viji Samuel Thomson, John Jose E, Ommen K. George

**Affiliations:** Department of Cardiology, Christian Medical College and Hospital, Vellore, India

**Keywords:** coiling, coronary angiogram, covered stent, percutaneous coronary intervention, pericardiocentesis

## Abstract

**Background:**

Coronary artery perforation (CAP) is an uncommon but serious complication of percutaneous coronary intervention, with a mortality rate of 10% to 20%. Its relevance is increasing with procedural complexity and expanded device use.

**Case Summary:**

We present a 10-year, single-center case series of 21 CAP cases, highlighting 5 key cases: type III CAP treated with a covered stent complicated by side branch occlusion; calcified perforation complicated by stent thrombosis; distal wire perforation with fatal outcome; chronic total occlusion-related CAP managed with coiling and surgery; and type IV CAP with delayed deterioration. Overall, type III CAP was most common (52%), and the left anterior descending artery was most frequently involved (57%). Management included covered stent in 71%, coiling in 14%, and surgery in 2 cases. Four patients died, and 2 patients developed stent thrombosis or in-stent restenosis.

**Discussion:**

This series emphasizes CAP mechanisms; treatment strategies; and the importance of timely recognition, imaging guidance, and institutional preparedness.

## Graphical Abstract

**Figure:**
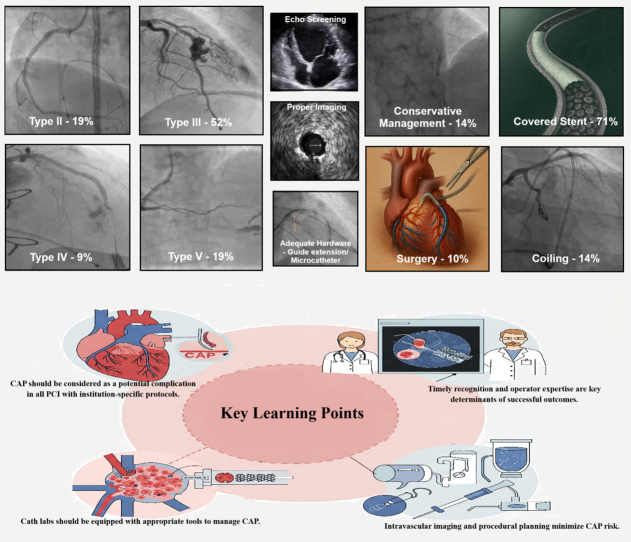

Percutaneous coronary intervention (PCI) is the standard treatment for coronary artery disease (CAD). Coronary artery perforation (CAP) is a rare but life-threatening complication of PCI, with an incidence of 0.1% to 0.6% and mortality ranging from 8% to 15%.^1^ The increase in complex PCIs, calcium modification tools, and aggressive dilations has increased the incidence of CAP. CAP is traditionally categorized into 4 types using the Ellis classification (Supplemental Table 1). Type I is the most common type. CAP types I and II are usually benign. Type III is more severe and often leads to cardiac tamponade requiring urgent pericardiocentesis. Type IV involves contrast leakage into a cardiac chamber and is managed similarly to type III. Some authors classify distal wire perforations as type V.^2^ Although initially subtle, these perforations can result in delayed tamponade requiring intervention.

At our institute, we perform about 2,000 PCIs annually, with CAP occurring in 2 to 5 cases (0.1%-0.3% incidence). We analyzed CAP cases over the past 10 years and collected comprehensive data for 21 patients. We highlight 5 representative cases here, each with distinct management and outcomes. Table 1 outlines management rationale, procedural challenges, potential advantages of newer technologies, and key lessons (timelines in Supplemental Table 2). All 21 cases are summarized in Table 2.

**Table 1:** Strategies, Challenges, Preventive Measures, and Key Lessons From 5 CAP Cases

Case	Management Strategies With Rationale	Intraprocedure Challenges and Resolutions	Planning Errors and Preventive Measures (Current-Day Practice)	Key Learning Points
1	• CS was deployed to seal LAD perforation owing to persistent extravasation. • Fenestration of CS to access LCx was considered but not performed owing to technical complexity.	LCx occlusion post-CS deployment: Managed conservatively, given stable vitals and retrograde filling.	1. Despite IVUS use, landing zone misjudgment led to vessel injury, highlighting the need for precise stent and balloon positioning to prevent perforation, especially near bifurcations. 2. Use of newer POT balloons with minimal shoulder overhang could have prevented this complication. 3. Use of a dual-lumen catheter could facilitate fenestration for side branch access while preserving main branch access.	1. Pre-procedural imaging alone is insufficient—accurate interpretation and precise application, especially in complex lesions and bifurcation zones, are essential to avoid perforation. 2. Optimal balloon selection is essential; dedicated or low-overhang POT balloons reduce inadvertent dilation, especially in bifurcation zones where oversizing must be avoided. 3. CSs risk side branch occlusion; dual-lumen catheters can be used for fenestration and side branch access if CS use is unavoidable, especially in LM/LAD lesions.
2	• CS was deployed to seal RCA perforation, which persisted despite balloon tamponade. • DES was used to reline stents post-subacute ST to restore patency and reduce rethrombosis risk from layered thrombus.	CS failed to track initially; pre-scaffolding with a DES facilitated successful delivery and deployment of the CS.	1. Perforation resulted from overdilatation of undermodified calcium. Calcium modification should be optimized based on early imaging findings, and aggressive dilation of calcified lesions must be avoided, even with imaging guidance. 2. The use of a low-profile CS such as PK-Papyrus enhances deliverability. 3. High-pressure NC post-dilation ensures optimal stent expansion, reducing the risk of ST secondary to CS.	1. Inadequate debulking and aggressive post-dilation markedly increase perforation risk in calcified lesions, even with imaging. Lesion preparation and device sizing must be guided by accurate imaging interpretation. 2 Orbital atherectomy is an effective strategy for treating heavily calcified ostial lesions. 3. If CS cannot cross, prelining with DES can aid successful tracking and delivery. 4. DES relining of CS can be an effective bailout in select cases.
3	• Coiling was considered owing to side branch involvement, where CS deployment was not feasible. • Surgery was considered later owing to hemodynamic instability.	The leak source remained unidentified angiographically, despite sealing the wire perforation; surgical exploration was considered.	1. Distal wire position must be closely monitored angiographically; advancing hydrophilic wires into small, tortuous branches without clear visualization increases perforation risk. 2. Early use of contrast echo or cardiac CT helps detect myocardial rupture when an angiogram is inconclusive. 3. If myocardial rupture is suspected, urgent surgical intervention should be prioritized.	1. Hydrophilic wires should be used cautiously and only with clear angiographic guidance. Contrast injections or microcatheters aid safe placement, and wire tip position must be continuously monitored. 2. Suspect myocardial rupture if effusion persists despite angiographic sealing; early imaging and surgical involvement may improve outcomes in such scenarios.
4	• Coiling was considered owing to side branch involvement, where CS deployment was not feasible. • Surgery was considered later owing to the persistence of extravasation despite coiling.	Persistent extravasation from inaccessible retrograde microcollaterals; surgical exploration was considered.	1. Intravascular imaging was not used to confirm vessel architecture or true lumen entry during CTO crossing. This could have guided safer wiring, appropriate balloon sizing. Low-profile IVUS catheters such as Philips Refinity or OptiCross offer better deliverability in such lesions. 2. Balloon pre-dilation in subintimal space should follow confirmed distal true lumen entry.	1. In CTOs, pre-dilation in ambiguous segments should be guided by imaging to confirm true lumen entry and lesion morphology, enabling safe wire positioning and accurate sizing. 2. Retrograde filling can cause delayed extravasation; continuous monitoring for late leaks is essential in CTO cases. 3. Early surgical involvement played a pivotal role in securing a favorable outcome in this case.
5	CS was preferred over coiling because of the risk of coil migration into the ventricular cavity.	CS delivery failed despite GuideLiner support; managed conservatively owing to stable vital signs and good distal flow.	1. In the absence of imaging, balloon oversizing and aggressive post-dilatation of undermodified calcium led to perforation. Imaging could have guided proper calcium modification and optimal device size. 2. Better guide support, anchoring techniques, and use of low-profile CS may have improved success. 3. A properly sized coil may have sealed the type IV leak effectively without migration. 4. Early recognition of infection in post-PCI patients with support devices is critical. Timely antibiotics, infectious work-ups, and early device removal might have prevented sepsis.	1. Imaging is essential for lesion preparation and device sizing, particularly in calcified lesions where caution post-dilatation is vital. 2. Incomplete lesion preparation and inappropriate stent or balloon sizing in calcified, tortuous vessels are strong predictors of perforation. 3. Initial hemodynamic stability can be misleading under IABP support. 4. If CS delivery fails, consider alternative strategies or early surgical referral. 5. Prolonged use of support devices such as IABP heightens the infection risk; early removal and timely infection management help prevent sepsis and multiorgan failure.

CAP = coronary artery perforation; CS = covered stent; CT = computed tomography; CTO = chronic total occlusion; DES = drug-eluting stent; IABP = intra-aortic balloon pump; IVUS = intravascular ultrasound; LAD = left anterior descending artery; LCx = left circumflex artery; LM = left main; NC = non-compliant; PCI = percutaneous coronary intervention; POT = proximal optimization technique; RCA = right coronary artery; ST = stent thrombosis.

**Table 2:** Summary of 21 Cases

Patient #	Age, y/Sex	Indication	CAP Type	Mechanism (Special Tools)	Bailout Strategy	Complication	Outcome
1	54/F	SIHD	III	NCB overexpansion at ostial LAD (IVUS)	CS	LCx occlusion	Stable, 4 y
2	73/F	STEMI	III	NCB overexpansion in calcified RCA (orbital + IVUS)	CS	Subacute ST	Stable, 1 y
3	59/M	SIHD	V	Wire exit in OMA (Sion Blue)	Coil + surgery	Ventricle rent	Died, 6 h
4	52/M	SIHD	III	SCB overexpansion in OMA CTO	Coil + surgery	Delayed leak	Stable, 4 y
5	72/M	NSTEMI	IV	High-pressure stenting in calcified/tortuous LAD (IABP)	W&W	MOF	Died, day 10
6	63/M	SIHD	III	NCB overexpansion in calcified RCA	CS	ISR	Stable, 3 y
7	62/F	SIHD	V	Wire exit in RCA (Fielder)	Coil	TIMI flow grade I	Stable, 2 y
8	67/F	SIHD	III	NCB overexpansion in tortuous LAD	CS (ping-pong)	None	Stable, 1 y
9	69/M	NSTEMI	V	Wire exit in LCx (Sion Blue)	W&W	None	Stable, 3 y
10	59/M	SIHD	II	NCB overexpansion in RCA biovascular scaffold (OCT)	CS	None	Stable, 2 y
11	53/M	SIHD	IV	SCB overexpansion in tortuous LAD (IVUS)	CS	Edge dissection	Stable, 1 y
12	77/M	SIHD	III	SCB overexpansion in calcified LCx (IABP)	CS	Arrest	Died, 3 h
13	66/F	SIHD	III	High-pressure stenting in calcified LAD	CS	None	Stable, 7 y
14	62/M	SIHD	III	SCB overexpansion in calcified LAD	CS	None	Stable, 10 y
15	50/M	STEMI	V	Wire exit in LAD (Fielder)	W&W	None	Stable, 10 y
16	50/M	NSTEMI	III	NCB overexpansion in LAD-D1 (IABP)	W&W	None	Stable, 10 y
17	78/F	STEMI	II	SCB overexpansion in RCA	CS	None	Stable, 1 y
18	69/F	SIHD	III	NCB overexpansion in LAD	CS	None	Stable, 1 y
19	70/F	NSTEMI	II	SCB overexpansion in distal LAD	CS	Arrest	Died, 1 h
20	53/F	SIHD	II	High-pressure stenting in RCA CTO	CS	None	Stable, 1 y
21	63/F	SIHD	III	NCB overexpansion in LAD CTO	CS (ping-pong)	None	Stable, 1 y

CAP = coronary artery perforation; CS = covered stenting; CTO = chronic total occlusion; D1 = first diagonal branch; F = female; IABP = intra-aortic balloon pump; ISR = in-stent restenosis; IVUS = intravascular ultrasound; LAD = left anterior descending artery; LCx = left circumflex artery; M = male; MOF = multiorgan failure; NCB = noncompliant balloon; NSTEMI = non–ST-segment elevation myocardial infarction; OCT = optical coherence tomography; OMA = obtuse marginal artery; RCA = right coronary artery; SCB = semicompliant balloon; SIHD = stable ischemic heart disease; ST = stent thrombosis; STEMI = ST-segment elevation myocardial infarction; TIMI = Thrombolysis In Myocardial Infarction; W&W = wait and watch.

## Case 1

**Figure 1:**
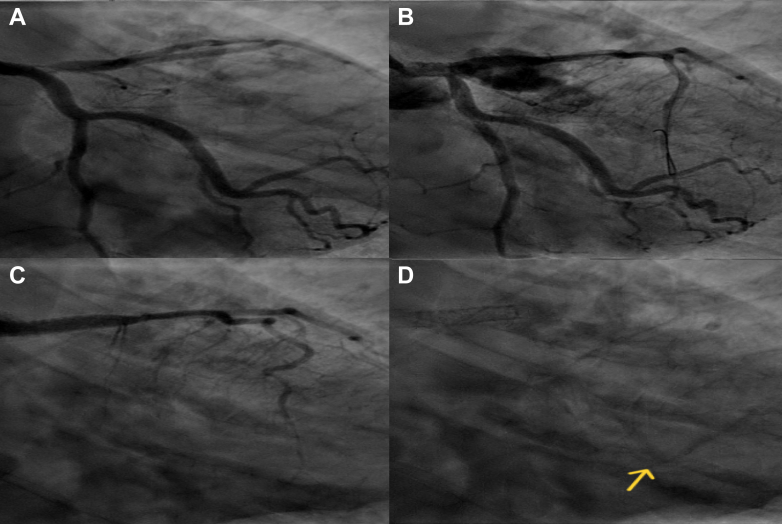
Angiography of Case 1 (A to D) Serial coronary angiographic images demonstrating progression and management of case 1. (A) Initial angiogram displaying a tight ostial lesion in the left anterior descending artery (LAD). (B) Type III coronary artery perforation post-dilatation of the left main coronary artery to LAD stent. (C) Left main coronary artery to LAD covered stent with absent flow in the left circumflex artery. (D) Late filling of obtuse marginal artery through retrograde channels (arrow).

A 54-year-old woman underwent coronary angiography for exertional angina, which revealed single-vessel CAD with a complex lesion in the ostioproximal left anterior descending artery (LAD). After intravascular ultrasound assessment, a 4.0 × 16 mm drug-eluting stent (DES) was deployed from the left main coronary artery to LAD. Post-dilation of the left main coronary artery to 5 mm inadvertently dilated the LAD ostium, resulting in an Ellis type III CAP. Emergency pericardiocentesis with autotransfusion was performed. A 3.5 × 16 mm Graftmaster covered stent (CS) (Abbott Medical) was deployed and post-dilated up to 4 mm. Post-stenting angiography showed no extravasation with Thrombolysis In Myocardial Infarction flow grade 3 into the LAD. However, there was no flow across the left circumflex artery (LCx). The patient remained hemodynamically stable despite a large territory of ischemia, with partial filling of the distal obtuse marginal artery (OMA) branch from the distal LAD on angiography performed 10 minutes after CS deployment. Although fenestration of the CS was considered, the patient was managed conservatively owing to hemodynamic stability after 30 minutes and the unavailability of electrocautery at the time. The patient was discharged on day 4 and remained well at 4-year follow-up, with echocardiography showing mild systolic dysfunction but no LCx abnormality (Figures 1A to 1D, Video 1).

## Case 2

**Figure 2:**
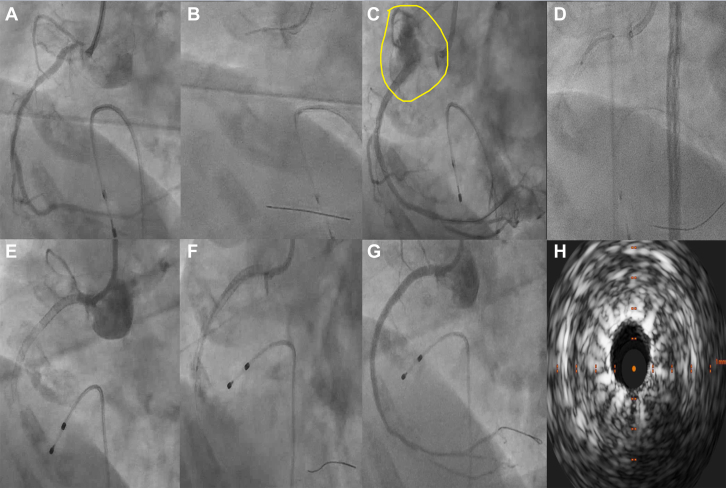
Angiography of Case 2 (A to G) Serial angiographic images illustrating lesion management; (H) intravascular ultrasound image of covered stent. (A) Baseline angiogram showing calcified, diffusely diseased right coronary artery with a tight ostial lesion. (B) Lesion prepared using orbital atherectomy. (C) Type III coronary artery perforation post-dilatation (marked circle). (D) Covered stent was deployed across the perforation using a GuideLiner support. (E) Subacute stent thrombosis at 48 hours. (F) Relining with an additional drug-eluting stent. (G) Final angiogram shows Thrombolysis In Myocardial Infarction flow grade III. (H) Intravascular ultrasound showing well-expanded stent struts with a sunburst appearance, a novel covered stent imaging sign.

A 73-year-old woman with a history of cerebrovascular accident 3 months ago presented with inferior wall myocardial infarction. Coronary angiography revealed calcific triple-vessel CAD, with a tight ostial right coronary artery (RCA) lesion. The lesion was resistant to high-pressure dilatations, and a 2-mm noncompliant balloon failed to track down the vessel. After 5 hours of unsuccessful attempts, PCI was abandoned, and the patient was advised to undergo coronary artery bypass graft surgery. Surgery was deemed high risk because of the patient’s recent stroke, and the surgical team recommended PCI instead. Given the significant ostial calcium, orbital atherectomy with retrograde sanding was considered the optimal strategy. She underwent PCI 2 weeks later with orbital atherectomy under intravascular ultrasound guidance. The mid-RCA was initially stented, and the proximal RCA was post-dilated using the same stent balloon. This resulted in a grade III CAP at the stent edge. Owing to failure in tracking a Graftmaster CS across the lesion, a DES was initially deployed using Guidezilla (Boston Scientific) support to scaffold the vessel and facilitate subsequent CS delivery. Two days later, repeat angiography for angina revealed RCA stent occlusion. Revascularization followed by intravascular ultrasound assessment identified incomplete apposition of the CS as the likely cause of stent thrombosis. Whole RCA stents were relined with another DES, achieving good flow down the vessel. PCI for the remaining vessels was planned later, and she was discharged after a week. The patient was doing well clinically at 1-year follow-up (Figures 2A to 2H, Video 2).

## Case 3

**Figure 3:**
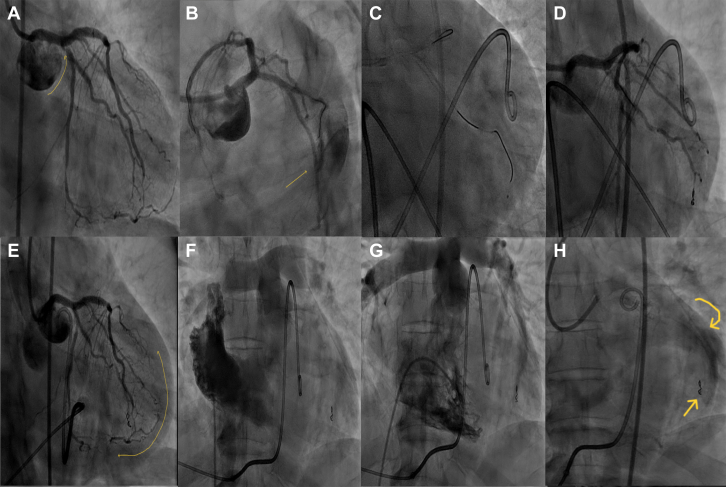
Angiography of Case 3 (A to H) Serial coronary angiographic images detailing case 3 progression and management. (A) Baseline angiogram shows a significant left circumflex artery ostial lesion (arrow). (B) Post-stenting, distal wire perforation into the obtuse marginal artery is seen. (C) Coils delivered via a Cantata Microcatheter (Cook). (D) Immediate good angiographic result after coiling. (E) Gradual pericardial fluid accumulation (double-ended arrow). (F and G) Right atrial and ventricular angiograms show normal right-sided flow. (H) Delayed angiogram reveals contrast material pooling over the lateral left ventricular wall (curved arrow) and embolization coil in the obtuse marginal artery (straight arrow).

A 59-year-old man who had PCI to the left main coronary artery, LAD, and LCx-OMA bifurcation 1 year ago presented with exertional angina. Coronary angiography revealed 80% in-stent restenosis of the LCx-OMA stent. A DES was deployed to cover the stenotic segment. Post-deployment angiography revealed a distal wire perforation of the OMA branch (Sion Blue; Asahi Intecc). After emergency pericardiocentesis with autotransfusion, a Tornado Embolization Coil (Cook Medical) was placed in the OMA branch to seal the perforation. Pericardial collection persisted despite successful sealing of the perforation. Aspirated fluid analysis showed low oxygen saturation, suggesting a venous source; however, right heart angiography did not reveal any perforation. Because of persistent effusion, the patient was transferred for emergency surgery. Sternotomy exposed a large infarct with a ventricular wall rupture, causing continuous extravasation, which was surgically repaired. Despite all efforts, the patient experienced cardiac arrest 3 hours post-surgery and could not be revived (Figures 3A to 3H, Video 3).

## Case 4

**Figure 4:**
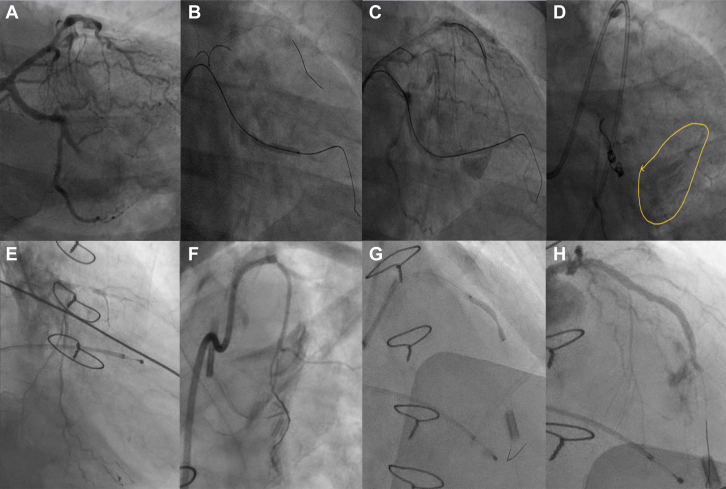
Angiography of Cases 4 and 5 (A to D) Serial coronary angiographic images of case 4. (E to H) Serial coronary angiographic images of case 5. Case 4: (A) Baseline angiogram shows chronic total occlusion of the obtuse marginal artery. (B) Lesion pre-dilated after successful chronic total occlusion wire crossing. (C) Type III coronary artery perforation post-dilatation. (D) Retrograde filling of the obtuse marginal artery post-coiling (marked circle). Case 5: (E) Baseline angiogram shows a significant left anterior descending artery lesion. (F) Failed attempt to revascularize the left internal mammary artery. (G) Stent deployed in mid–left anterior descending artery with unyielded lesion. (H) Type IV coronary artery perforation post-stenting.

A 52-year-old man with a history of myocardial infarction 10 years ago underwent coronary angiography for angina, which revealed a critical lesion in the diagonal branch and chronic total occlusion of the second OMA branch. Following balloon angioplasty of the diagonal branch, the OMA branch was recanalized and dilated using a 2.5-mm semicompliant balloon at 12 atm. Post-dilatation angiography revealed a grade III CAP. Emergency pericardiocentesis with autotransfusion was performed, and the perforation was sealed using a Tornado Embolization Coil. Although the extravasation was temporarily stopped, the patient's condition deteriorated 2 hours after the procedure. Repeat angiography demonstrated late filling of the OMA branch, distal to the coils, via a retrograde channel, with persisting extravasation. Given the ongoing leak, an emergency sternotomy was performed to surgically repair the OMA vessel. The patient was discharged on the 10th day and remained clinically stable during 4-year follow-up (Figures 4A to 4D, Video 4).

## Case 5

A 72-year-old man with a history of coronary artery bypass graft surgery with 4 grafts 15 years ago presented with acute chest pain and unresponsiveness. After resuscitation, he underwent PCI with intra-aortic balloon pump support. Coronary angiography revealed native triple-vessel CAD, patent venous grafts to RCA and distal LAD, occluded venous graft to ramus intermedius, and left internal mammary artery graft to LAD. Attempts to stent the ramus intermedius and recanalize the arterial graft were unsuccessful, so PCI was performed on the native LAD using 2 DESs (2.75 × 36 mm and 2.5 × 19 mm). The distal stent was deployed at high pressure owing to a resistant calcified lesion. Post-stenting angiography revealed perforation at the distal edge of the stent with extravasation into the left ventricle. Multiple attempts to deliver a Graftmaster CS using GuideLiner (Teleflex) support were unsuccessful. Given the presence of Thrombolysis In Myocardial Infarction flow grade 3 in the vessel and the patient's initial hemodynamic stability, further intervention was not pursued. The patient gradually deteriorated in the intensive care unit, developing sepsis and acute renal failure. Despite supportive care, he died after 10 days from multiple organ dysfunction (Figures 4E to 4H, Video 5).

## Discussion

**Figure 5:**
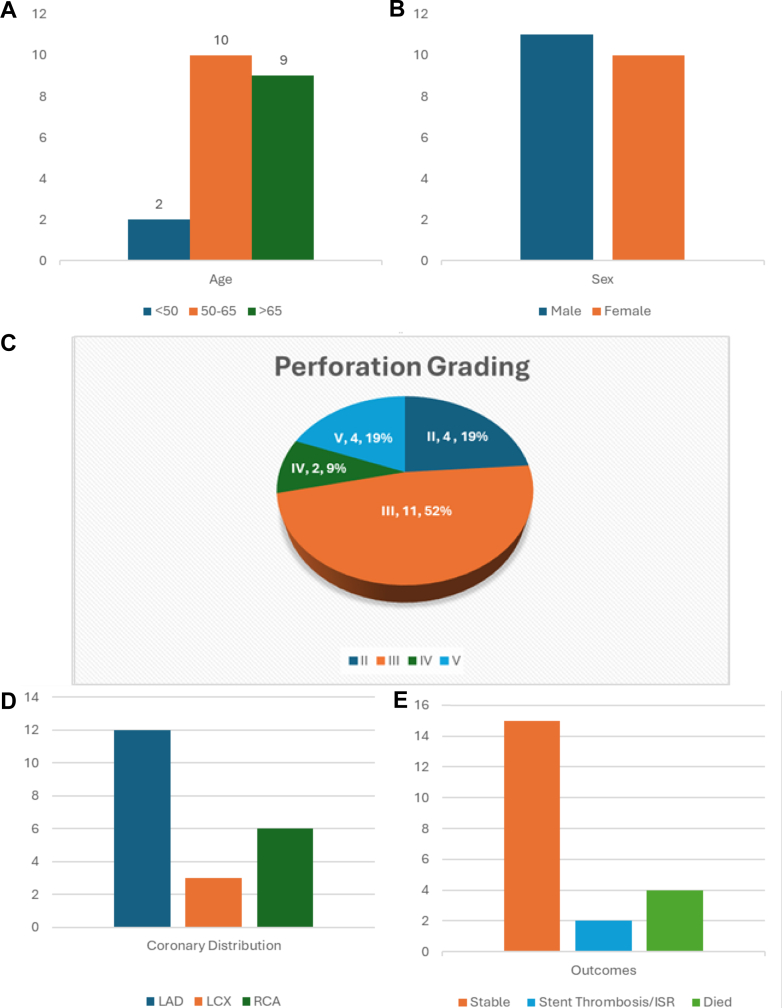
Comparative Analysis of Age, Sex, Perforation Grading, Coronary Distribution, and Outcomes in 21 Cases Demographic and procedural characteristics of patients with coronary artery perforation. (A, B, D, and E) Bar charts depicting age distribution, sex distribution, coronary artery involvement, and patient outcomes among the cases. (C) Pie chart illustrating the classification of coronary perforations based on severity grading (Ellis classification). ISR = in-stent restenosis; LAD = left anterior descending artery; LCX = left circumflex artery; RCA = right coronary artery.

Timely recognition and skilled management are crucial in determining CAP outcomes. Incidence and prognosis vary across regions owing to differences in health care systems, procedural techniques, and reporting practices. This case series captures the full spectrum of CAP presentations and provides a concise overview of real-world management strategies. Among the 21 cases analyzed (Figure 5, Table 2), 90% of patients were older than 50 years of age, with equal gender distribution. Type III CAP was the most common type (52%), and the LAD was the frequently involved vessel (57%). CSs were used in 71% of cases, coiling and conservative management were used in 14% each, and surgery was performed in 9.5%. Overall mortality was 19%, and 9.5% experienced stent thrombosis or restenosis. Five representative cases are highlighted to illustrate key patterns.

**Figure 6:**
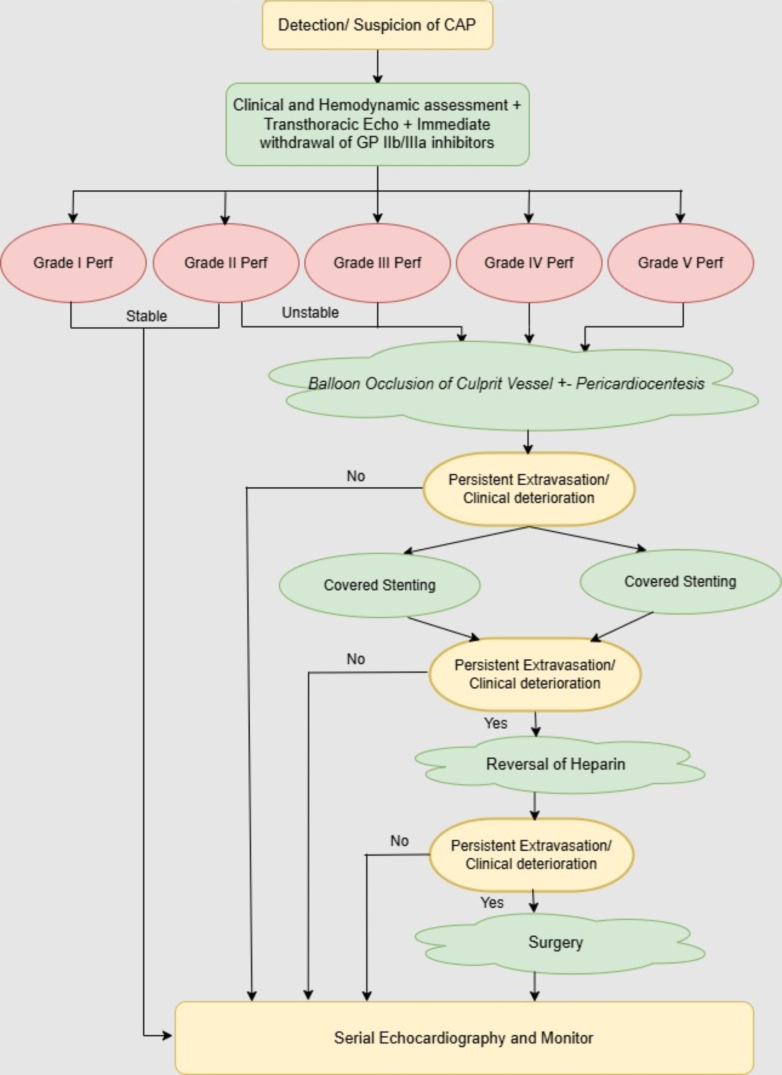
Coronary Artery Perforation Management Algorithm Flowchart outlines the stepwise approach to diagnosing and managing coronary artery perforation encountered during percutaneous coronary intervention. Management depends on the severity (Ellis classification), hemodynamic status, and angiographic features and may include prolonged balloon tamponade, reversal of anticoagulation, use of covered stents, embolization techniques, and surgical intervention. CAP = coronary artery perforation; GP = glycoprotein; Perf = perforation.

Management of CAP includes anticoagulation reversal, balloon tamponade, CSs, coil embolization, and surgery (Figure 6). Strategy selection depends on the perforation site and severity, hemodynamic status, and resource availability. Given the potential for rapid deterioration, a multidisciplinary approach with early surgical consultation and critical care preparedness is essential. Small perforations often seal with submaximal pressure balloon inflations for 5 to 10 minutes above the rupture site while being mindful of distal vessel ischemia and arrhythmias (cases 9, 15, and 16).^3^ Newer perfusion balloons have been developed to seal the perforation site while maintaining distal flow.^4^ If unsuccessful, prompt pericardiocentesis with drain placement is warranted. Substantial pericardial fluid should be drained before anticoagulation reversal to prevent tamponade progression. If necessary, anticoagulation can be reversed with protamine, glycoprotein IIb/IIIa inhibitors can be discontinued, and platelet transfusions can be considered. Protamine reversal should be performed only after withdrawing all intracoronary hardware, as premature reversal increases the risk of acute thrombosis or device entrapment. The association between glycoprotein IIb/IIIa inhibitors and CAP remains controversial, possibly due to selection bias in complex cases or the potential for minor tears to develop into serious perforations.^5^ Type III CAP carries higher mortality than type IV CAP owing to frequent cardiac tamponade.^1^^,^^6^ Immediate bedside echocardiography is essential for effusion assessment, whereas serial scans aid in the detection of delayed effusions from missed leaks and in monitoring existing collections.

The CS serves as a cornerstone in managing major perforations (cases 1, 2, 6, 8, 10-14, 17-21; Videos 1, 2, 6, and Video 8, Video 9, Video 10). Nevertheless, their use carries distinct risks, including side branch occlusion (case 1; Video 1), stent thrombosis (cases 2 and 13; Videos 2 and 9), and in-stent restenosis (case 6; Video 6).^7^ Fenestration of the CS using electrocautery-assisted reentry may be considered in large or flow-limiting side branch occlusions. In case 1, the patient remained hemodynamically stable with retrograde OMA filling, and electrocautery was unavailable at the time; otherwise, fenestration would have been attempted. In this series, only Graftmaster and pro-graft stents were used. Although effective, their bulky design limits deliverability in complex anatomies such as tortuous, angulated, or calcified vessels (case 5). Newer-generation CSs, such as PK-Papyrus and Be-Graft, with thinner struts and better flexibility, may simplify CAP management in complex anatomies. Retrieving the balloon and inserting a CS may be time-consuming. The CS can be delivered using the same guide system or through a ping-pong technique using an alternate guide system (cases 8 and 21) (Video 10).^8^ Guide extension catheters can improve delivery success (cases 2 and 11). Owing to higher risks of thrombosis and restenosis, high-pressure post-dilation is essential even after sealing the perforation.

Coil embolization via microcatheters is effective for distal or side branch perforations (cases 3, 4, and 7; Videos 3, 4, and 7), although efficacy is limited by coil-vessel size mismatch. Early-generation fibered coils used wool and Dacron, with Dacron showing better platelet aggregation and lower inflammation. Newer coils (hydrogel-coated and bare platinum) achieve hemostasis primarily through mechanical occlusion, making them ideal in coagulopathic patients with coagulopathy. Hydrogel coils expand on blood contact, enhancing packing density without relying on thrombosis, whereas bare platinum coils provide circumferential mechanical occlusion. Although most coils require 0.018-inch microcatheters, some neurovascular systems such as Axium and Concerto (both Medtronic) are compatible with 0.014-inch coronary microcatheters, enabling distal delivery without upsizing the catheter.^9^ Alternative sealing methods include thrombin injection, autologous blood clots, and fat embolization.^10^ All of these methods pose risks of vessel occlusion and possible infarction (case 3) and should be used only when the risk of myocardial injury is low. In cases of severe ischemia, hemodynamic instability, or unsealed or unidentified perforations, emergency surgery is imperative (cases 3 and 4). Although surgery is pivotal in saving lives, it carries higher risks of morbidity and mortality.

CAP prevention relies on thorough preprocedural planning, careful wire manipulation, and conservative ballooning. Individualized risk assessment, intravascular imaging, and meticulous lesion preparation can reduce CAP incidence and severity. Intravascular imaging is essential in complex PCI, particularly before atherectomy or high-pressure dilatation, for accurate vessel sizing, calcium assessment, and guiding lesion preparation. Imaging confirms intraluminal wire position; identifies subtle dissections or intramural hematomas not evident on angiography; and, importantly, differentiates concentric from nodular calcium, the latter carrying higher perforation risk owing to its protrusive morphology. Effective vessel preparation is crucial in calcified lesions, and using slightly undersized high-pressure balloons may reduce perforation risk when ultra-high pressures are required. Limited imaging access a decade ago may have contributed to otherwise avoidable perforations. Institutional preparedness, including predefined CAP protocols, availability of CSs and coils, and early surgical backup, remains essential to mitigate impact and improve outcomes.

## Conclusions

CAP, though rare, remains a serious complication of PCI, particularly in complex and calcified lesions. Our 10-year case series highlights the diverse presentations and management strategies of CAP, emphasizing the importance of early recognition, tailored treatment, institutional preparedness, and multidisciplinary coordination. Intravascular imaging aids in prevention and precise management by guiding lesion preparation and device sizing. Routine imaging and increased operator awareness in high-risk PCI can reduce CAP incidence and improve outcomes.

**Visual Summary:**
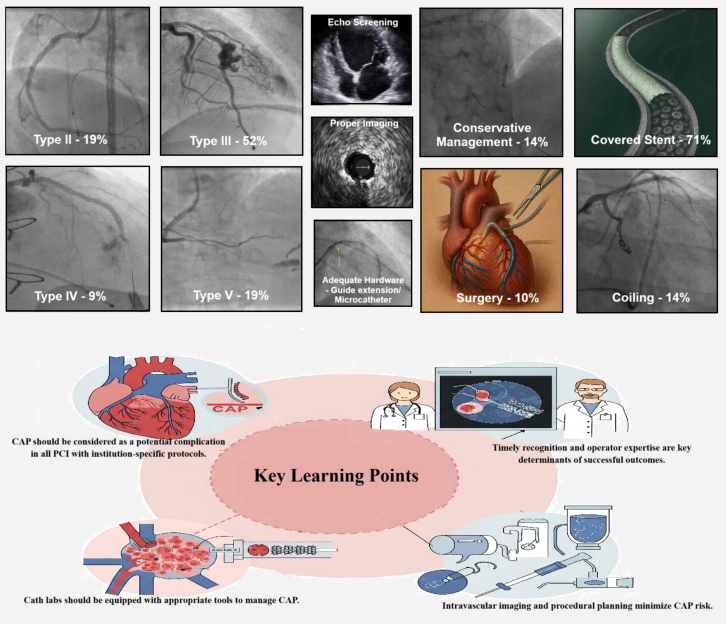
Coronary Artery Perforation

## Funding Support and Author Disclosures

The authors have reported that they have no relationships relevant to the contents of this paper to disclose.

**Take-Home Messages**

• CAP should be considered as a potential complication of all PCI cases, requiring individualized and institution-specific management protocols.
• Operator expertise, timely recognition, and the use of intravascular imaging for procedural planning are crucial to minimizing risk and improving outcomes.
• All catheterization laboratories should be equipped with embolization coils, CSs, specialized catheters for delivering CSs, and perfusion balloons to manage CAP cases effectively.

## Appendix

Video 1 Left Main Coronary Artery to LAD Stenting Complicated by Balloon-Induced Perforation in Case 1 Angiogram showing a significant lesion in the ostium of the left anterior descending artery (LAD) at presentation. After intravascular ultrasound assessment, the left main coronary artery to LAD was stented with a drug-eluting stent. Accidental slippage of the balloon during post-dilatation of the left main coronary artery part caused grade III perforation, sealed with a covered stent, compromising left circumflex artery flow. The subsequent angiogram obtained after 10 to 15 minutes revealed a partially filling obtuse marginal branch of the LCx artery.

Video 2 Calcified RCA Ostial Lesion Treated With Atherectomy, Ending in Perforation and Stent Thrombosis of Covered Stent in Case 2 Tight right coronary artery (RCA) ostial lesion treated with orbital atherectomy after multiple failed attempts. Balloon dilatation of the proximal RCA, after stenting mid-RCA, resulted in grade III perforation. A drug-eluting stent is used for initial sealing, followed by a covered stent via GuideLiner support. Subacute stent thrombosis is managed by relining with another drug-eluting stent.

Video 3 Wire-Induced Perforation Post-Stenting of LCx In-Stent Restenosis in Case 3 In-stent restenosis in proximal left circumflex artery (LCx) treated with DES. Post-stenting angiogram revealed wire-induced perforation from the obtuse marginal artery branch. Coil embolization is attempted, but the leak persists, which was not evident even after right-sided angiography.

Video 4 Chronic Total Occlusion Recanalization Complicated by OMA Perforation in Case 4 Angiogram shows chronic total occlusion of obtuse marginal artery (OMA) and lesion in the diagonal branch. OMA was recanalized after plain balloon angioplasty of the diagonal branch. Subsequent OMA ballooning causes grade III perforation, which persists despite successful coiling.

Video 5 Grade IV Perforation After LAD Stenting in a Patient Post-Coronary Artery Bypass Graft in Case 5 The Angiographic video of case 5 shows a native triple-vessel coronary artery disease with an atretic left internal mammary artery graft. Stenting of the left anterior descending artery (LAD) resulted in grade IV perforation from the distal edge of the stent.

Video 6 RCA Perforation Sealed with Covered Stent; Restenosis at 4-Year Follow-Up in Case 6 Angiogram shows a diffusely diseased right coronary artery (RCA). Stenting and post-dilatation resulted in grade III perforation, successfully sealed with a covered stent after proximal balloon occlusion and pericardiocentesis. Angiogram at 4-year follow-up shows significant in-stent restenosis at the stent junction; ballooning of the lesion restores flow.

Video 7 Wire-Induced Posterior Descending Artery Perforation Managed by Coil Embolization in Case 7 Right coronary artery stenting followed by wire-induced perforation in posterior descending artery. Coil embolization achieves initial Thrombolysis In Myocardial Infarction flow grade 1, improving to Thrombolysis In Myocardial Infarction flow grade 3 on follow-up angiogram.

Video 8 LCx Perforation Post-Stenting in Case 12 Significant left main coronary artery and triple-vessel coronary artery disease. Left circumflex artery (LCx) post-dilatation results in grade III perforation, successfully sealed with a covered stent, restoring vessel flow.

Video 9 LAD Perforation From Distal Stent Edge Managed With Covered Stent in Case 13 Left anterior descending artery (LAD) stenting causes grade III perforation from the distal edge. Temporary balloon occlusion seals the leak. LAD is occluded at 90 minutes follow-up. Sealing of the perforation site with a covered stent resulted in good flow down the vessel.

Video 10 LAD Perforation Sealed via Dual Catheter Approach in Case 21 Proximal left anterior descending artery (LAD) lesion treated with a stent post-dilatation led to grade III perforation. Balloon occlusion and dual-guiding catheter technique used for covered stent delivery. Final stenting resulted in good flow down the vessel.

Supplemental Tables 1 Ellis Classification of Coronary Artery Perforation (1994) **Supplemental Table 2:** Timeline of Cases 1-5
