# Supplementary material for: Coronary Artery Perforation During PCI: Mechanisms, Management, and Outcomes From a 10-Year Experience
Source: JACC Case Rep. 2025 Sep 24;30(29):105068. doi: 10.1016/j.jaccas.2025.105068 (PMC12539445; doi:10.1016/j.jaccas.2025.105068)
Supplement: Supplemental Tables 1 — Ellis Classification of Coronary Artery Perforation (1994) Supplemental Table 2: Timeline of Cases 1-5 [file mmc11.docx]

Supplementary Table 1: Ellis Classification of Coronary Artery Perforation (1994)

| Type I (Concealed) | Extraluminal crater without extravasation |
| --- | --- |
| Type II  (Limited) | Pericardial or myocardial blush without contrast Jet extravasation |
| Type III  (Free flowing) | Extravasation jet through a frank perforation |
| Type IV  (Cavity spilling) | Perforation into an anatomic chamber, such as the coronary sinus, atria, or ventricles |
| Source: Ellis SG, et al. Circulation. 1994;90(6):2725–2730. doi:10.1161/01.cir.90.6.2725. | |

# Supplementary Table 2: Timeline of Cases 1-5

| \| Case 1 (54F, Exertional Angina) \| \| \| --- \| --- \| \| Day 0 \| Angiogram: Single-vessel CAD, complex ostio-proximal LAD lesion. PCI: Left main to LAD stenting. Complication: Type III CAP (LAD ostium). Management: Pericardiocentesis, autotransfusion, deployment of Graft-master CS.  Complication related to CS deployment: No flow in LCX \| \| 30 min post \| Stable–conservative management (no fenestration). \| \| Day 4 \| Discharged in stable condition. \| \| 4-Year F/U \| Clinically well. \| \| Case 2 (73F, Inferior MI with recent stroke) \| \| \| Day 0 \| Failed PCI to a tight calcific RCA lesion; CABG deferred due to recent stroke. \| \| 2 Weeks Later \| PCI with orbital atherectomy and Intravascular ultrasound. Complication: Type III CAP post-stenting. Management: Graft-master CS deployed. \| \| Day 2 \| RCA stent thrombosis – relined with another DES, flow restored. \| \| Day 7 \| Discharged in stable condition. \| \| 1-Year F/U \| Clinically stable. \| \| Case 3 (59M, Prior Left Main-LAD-LCX-OM PCI) \| \| \| Day 0 \| In-stent restenosis in LCX-OM stent – treated with DES. Complication: Type V CAP of OM. Management: Pericardiocentesis, autotransfusion, coil embolization. \| \| Post-PCI \| Persistent effusion; no clear leak source. \| \| Surgery \| Sternotomy: ventricular wall rent repaired. \| \| 3 hours post-surgery \| Cardiac arrest – not revived. \| \| Case 4 (52M, Prior MI) \| \| \| Day 0 \| Angiogram: Diagonal lesion + Chronic total occlusion of OM. PCI: Balloon angioplasty to the diagonal and OM. Complication: Type V CAP of OM. Management: Pericardiocentesis, autotransfusion, coil embolization. \| \| 2 hours later \| Persistent leak – emergency sternotomy to repair OM. \| \| Day 10 \| Discharged stable. \| \| 4-Year F/U \| Clinically stable. \| \| Case 5 (72M, Prior CABG, Acute MI) \| \| \| Day 0 \| Angiogram: Triple vessel disease  PCI: Failed PCI to Ramus/LIMA; followed by two DES to LAD. Complication: Type IV CAP post-dilatation. Management: Failed Graft-master delivery; no further intervention (TIMI III flow, stable). \| \| Days 1–3 \| ICU monitoring. \| \| Days 4–7 \| Clinical decline: sepsis + acute renal failure. \| \| Day 10 \| Death due to multi-organ dysfunction. \| |
| --- | --- | --- | --- | --- | --- | --- | --- | --- | --- | --- | --- | --- | --- | --- | --- | --- | --- | --- | --- | --- | --- | --- | --- | --- | --- | --- | --- | --- | --- | --- | --- | --- | --- | --- | --- | --- | --- | --- | --- | --- | --- | --- | --- | --- | --- | --- | --- | --- | --- | --- | --- | --- |
| CABG=Coronary Artery Bypass grafting, CAD=Coronary Artery Disease, CAP=Coronary Artery Perforation, CS=Covered stent, DES=Drug-eluting stent, F=Female, ICU=Intensive Care unit, LAD=Left Anterior Descending artery, LCX=Left Circumflex Artery, LIMA=Left Internal Mammary Artery, M=Male, MI=Myocardial Infarction, OM=Obtuse Marginal, RCA=Right Coronary Artery, TIMI=Thrombolysis In Myocardial Infarction score. |
